# Supplementary material for: Development of a computer-aided design software for the quantitative evaluation of aesthetic damage
Source: PLoS One. 2019 Dec 18;14(12):e0226322. doi: 10.1371/journal.pone.0226322 (PMC6919621; doi:10.1371/journal.pone.0226322)
Supplement: S1 Code site — 1. (ZIP) [file pone.0226322.s001.zip › [EESC_jr][Projeto]Relatorio_07_21/reports/templates_zip/gentelella-master.zip!/gentelella-master/production/index2.html]

Gentellela Alela! | 


Gentellela Alela!

Welcome,

## John Doe

  


### General

- Home 
  - Dashboard
  - Dashboard2
  - Dashboard3
- Forms 
  - General Form
  - Advanced Components
  - Form Validation
  - Form Wizard
  - Form Upload
  - Form Buttons
- UI Elements 
  - General Elements
  - Media Gallery
  - Typography
  - Icons
  - Glyphicons
  - Widgets
  - Invoice
  - Inbox
  - Calendar
- Tables 
  - Tables
  - Table Dynamic
- Data Presentation 
  - Chart JS
  - Chart JS2
  - Moris JS
  - ECharts
  - Other Charts
- Layouts 
  - Fixed Sidebar
  - Fixed Footer

### Live On

- Additional Pages 
  - E-commerce
  - Projects
  - Project Detail
  - Contacts
  - Profile
- Extras 
  - 403 Error
  - 404 Error
  - 500 Error
  - Plain Page
  - Login Page
  - Pricing Tables
- Multilevel Menu 
  - Level One- Level One
      - Level Two
      - Level Two
      - Level Two
    - Level One
- Landing Page Coming Soon

- John Doe
  - Profile
  - 50%
    Settings
  - Help
  - Log Out
- 6
  - John Smith
    3 mins ago

    Film festivals used to be do-or-die moments for movie makers. They were where...
  - John Smith
    3 mins ago

    Film festivals used to be do-or-die moments for movie makers. They were where...
  - John Smith
    3 mins ago

    Film festivals used to be do-or-die moments for movie makers. They were where...
  - John Smith
    3 mins ago

    Film festivals used to be do-or-die moments for movie makers. They were where...
  - **See All Alerts**


179

### New Sign ups

Lorem ipsum psdea itgum rixt.

179

### New Sign ups

Lorem ipsum psdea itgum rixt.

179

### New Sign ups

Lorem ipsum psdea itgum rixt.

179

### New Sign ups

Lorem ipsum psdea itgum rixt.

## Transaction Summary Weekly progress

December 30, 2014 - January 28, 2015

Total Sessions

## 231,809

Total Revenue

## $231,809

Total Sessions

## 231,809

## Top Profiles

- - Settings 1
  - Settings 2

- Ms. Mary Jane

  **$2300.**  Agent Avarage Sales

  12 Sales Today
- Ms. Mary Jane

  **$2300.**  Agent Avarage Sales

  12 Sales Today
- Ms. Mary Jane

  **$2300.**  Agent Avarage Sales

  12 Sales Today
- Ms. Mary Jane

  **$2300.**  Agent Avarage Sales

  12 Sales Today
- Ms. Mary Jane

  **$2300.**  Agent Avarage Sales

  12 Sales Today

## Weekly Summary Activity shares

- - Settings 1
  - Settings 2

#### Weekly sales progress

#### Bounce Rates

#### New Traffic

#### Device Share

## Top Profiles Sessions

- - Settings 1
  - Settings 2

April

23

Item One Title

Lorem ipsum dolor sit amet, consectetur adipiscing elit.

April

23

Item Two Title

Lorem ipsum dolor sit amet, consectetur adipiscing elit.

April

23

Item Two Title

Lorem ipsum dolor sit amet, consectetur adipiscing elit.

April

23

Item Two Title

Lorem ipsum dolor sit amet, consectetur adipiscing elit.

April

23

Item Three Title

Lorem ipsum dolor sit amet, consectetur adipiscing elit.

## Top Profiles Sessions

- - Settings 1
  - Settings 2

April

23

Item One Title

Lorem ipsum dolor sit amet, consectetur adipiscing elit.

April

23

Item Two Title

Lorem ipsum dolor sit amet, consectetur adipiscing elit.

April

23

Item Two Title

Lorem ipsum dolor sit amet, consectetur adipiscing elit.

April

23

Item Two Title

Lorem ipsum dolor sit amet, consectetur adipiscing elit.

April

23

Item Three Title

Lorem ipsum dolor sit amet, consectetur adipiscing elit.

## Top Profiles Sessions

- - Settings 1
  - Settings 2

April

23

Item One Title

Lorem ipsum dolor sit amet, consectetur adipiscing elit.

April

23

Item Two Title

Lorem ipsum dolor sit amet, consectetur adipiscing elit.

April

23

Item Two Title

Lorem ipsum dolor sit amet, consectetur adipiscing elit.

April

23

Item Two Title

Lorem ipsum dolor sit amet, consectetur adipiscing elit.

April

23

Item Three Title

Lorem ipsum dolor sit amet, consectetur adipiscing elit.


Gentelella - Bootstrap Admin Template by Colorlib
